# Is It Good Practice to Leave the Dentinal Tubuli Open?

**Published:** 1874-07

**Authors:** W. Storer How


### IS IT GOOD PRACTICE TO LEAVE THE DENTINAL TUBULI OPEN?

BY W. STORER HOW, D. D. S.

I have just been reading Dr. Hassell's article in the June Register, and I really feel almost like telegraphing in my anxiety to answer that question most emphatically in the negative; certainly not, my dear doctor, not by any means.

I do earnestly hope that in the case of "a small gold filling entirely surrounded by the mouths of dentinal tubuli considerably dilated," and "full of stinking matter," that the tocsin of the "litmus" brought all the forces of his mind into action and reaction until there were no more tubes left exposed to make mischief. The bare possibility, however, that practice may not have followed theory, impels me at once to suggest that "since it is the animal creation which completes the work of destruction," the first thing to be done is to stop this work of destruction, and of course the keen observer of those open mouths would soon become familiar with the habits of the animals so that properly baited traps could be set at such a distance from the mouths of the caves that the animals should have no support against which they might plant their feet and tear loose and break things; possibly some might be captured unhurt, or young, and trained to use, with pumps and cranes, in the removal of the stinking matter from the tubes, though such service would be quite unnecessary in a steam dental establishment, which would doubtless also utilize the animal's remains. Then, the application of disinfectants and the energetic use of something like boiler flue scrapers, adapted to the Morrison engine, accompanied with proper and prolonged systemic treatment, would leave the tubes in a condition to be separately closed after the preparation of suitable retaining points using octagon pellets, number seven hundred and fifty, well malleted in, and finished in oil.

In the case of recently exposed tubuli, the subject increases in interest and delicacy, for after securing the crop of parasites, it may be found expedient to perform flap operations on some of the fibrils, and also to employ several others of the remarkable methods known only to every dentist.

The concave cap having a central perforation 1-10,000 of an inch, will enable the operator to make exact contact with the healed fibril, (the flap cicatrix being visible through the perforation) and then by means of retaining screws, well hardened foil and a fourteen pound elastic mallet, every tube can be effectually closed. Indeed, on no account should any of them be left open, as any one will perceive, who can see the dilated mouths of the tubuli and observe the insatiate Leptothrix clawing away in and out.

It is not expected that these suggestions will be of any value to the ordinary practitioner, except to occasion surprise that any other dentist had ever thought out these things which have long been matters of common or discarded practice with him, and to prove this he would like to show you his "traps," but it is very confidently and even gloriously expected that by these or some other ways and means Dr. Hassell's question shall be negatively answered beyond question, thus putting a scientific and auriferous stop to the further ravages of those abominable oral parasites.

